# Utilities of 3D technology for planning endoscopic endonasal approaches. Case series of anatomical-imaging review

**DOI:** 10.1093/jscr/rjaf087

**Published:** 2025-03-09

**Authors:** Daniel Alejandro Vega-Moreno, Gervith Reyes-Soto, Monica Serrano-Murillo, Ulises García González, Oscar Medina-Carrillo

**Affiliations:** Neuro-oncology Unit, Instituto Nacional de Cancerología, Avenida San Fernando 22, Belisario Domínguez Sección 16, Tlalpan 14090, Ciudad de México, México; Postgraduated Division, Universidad Nacional Autónoma de México, Cto. de los Posgrados S/N, C.U., Coyoacán, 04510 Ciudad de México, CDMX México; Neuro-oncology Unit, Instituto Nacional de Cancerología, Avenida San Fernando 22, Belisario Domínguez Sección 16, Tlalpan 14090, Ciudad de México, México; Neuro-oncology Unit, Instituto Nacional de Cancerología, Avenida San Fernando 22, Belisario Domínguez Sección 16, Tlalpan 14090, Ciudad de México, México; Neurosurgery Department, Hospital Central Sur de Alta Especialidad, PEMEX. Anillo Perif. 4091, Fuentes del Pedregal, Tlalpan, 14140 Ciudad de México, Mexico; Neurosurgery Department, Hospital Central Sur de Alta Especialidad, PEMEX. Anillo Perif. 4091, Fuentes del Pedregal, Tlalpan, 14140 Ciudad de México, Mexico

**Keywords:** endoscopic endonasal, sellar tumor, 3D planning surgery, skull base surgery

## Abstract

Endoscopic endonasal surgery requires specific training and essential anatomical and technical knowledge. The support of 3D technologies favors the development of this knowledge. We exemplify the use of this 3D reconstruction tool through four clinical cases of sellar tumors. Imaging analysis was performed on four patients diagnosed with sellar tumors who underwent resection surgeries using endoscopic endonasal approaches with the support of 3D reconstructions. Four sellar tumors and their related anatomical structures were reconstructed using manual planimetric segmentation to modify the pre- and trans-surgical decision. Although 3D reconstruction images are not new, the imaging studies with which we can make these reconstructions have been improving and with which to improve anatomical and surgical understanding in endoscopic transnasal surgery. This work exemplifies the usefulness of 3D technology in planning endoscopic endonasal surgeries for the different tumor pathologies of the sellar region.

## Introduction

Endoscopic endonasal surgery requires specific training and necessary anatomical and technical knowledge. For this, multiple tools have been developed over time that favors training and training in this discipline. Animal models, cadavers, artificial models, and simulators have been used [1]. Magnetic resonance imaging is the study of choice for the diagnosis and surgical planning of pituitary adenomas [2]. And although its use is mandatory in most centers for the diagnosis and treatment of sellar tumors, attempts have been made to create new areas of work and uses of 3D technology. The use of this has been expanded to gastrointestinal surgeries, cardiac surgery, and even pituitary adenoma surgeries [3, 4]. 3D reconstruction images are predictors of an excellent surgical resection and, therefore, have been correlated with good post-operative results [5]. Furthermore, using this tool, it has been possible to evaluate areas with residual tumors and thus achieve higher rates of total resection [6]. Conversely, geometric volumetry is never exact, so 3D volumetry by segmentation is considered superior regarding volume measurement. So, we resort to manual volumetric segmentation whenever we want to know the exact total volume [7]. The surgeon must know the surgical anatomy; however, the planimetric imaging review entails certain difficulties. Therefore, this work tool gives surgeons a better understanding of the anatomy and the anticipation of scenarios and variants during surgery. We exemplify the use of this 3D reconstruction tool through four clinical cases in which the 3D reconstruction helped us make surgical decisions and improve individual anatomical knowledge, maximizing neuro-vascular compression and avoiding possible trans-surgical complications.

## Case series

Imaging analysis was performed on four patients with a diagnosis of sellar tumor who underwent resection surgeries using endoscopic endonasal approaches. These magnetic resonance images were collected in T1, T2, and contrast-enhanced T1 sequences and, in one case, 3D TOF. Manual planimetric segmentation in axial view was performed with the ITK-SNAP program (ITK-Snap, version 3.8.0, University of Pennsylvania). The image processing program automatically generated a 3D reconstruction. For segmentation, merging the different sequences and manually marking the critical anatomical structures of interest by voxels was necessary. Once the shading of all the structures of interest was done, the 3D modeling was carried out automatically, obtaining the reconstructions of each of the images of both the tumor and the vascular structures that we believed were important to pay attention to at the time of resection. These images were subsequently used transoperatively for surgery planning and decision-making. The demographic characteristics of the patients are shown in Table 1.

**Table 1:** Demographics of patients treated using endoscopic endonasal approaches with manual 3D segmentation support

**Number case**	**Age**	**Sex**	**Histopathological**	**Clinic**	**Utility 3D reconstruccion**
1	71	Female	Craniopharingioma	Visual disturbance and insipid diabetes	Avoid vascular lesion
2	42	Male	Pituitary adenoma	Visual disturbance and IHS	Volumetric measure
3	68	Male	Pituitary adenoma	Visual disturbance	Avoid vascular lesion
4	42	Male	Pituitary adenoma	Visual disturbance	Avoid vascular lesion

### Case 1

**Figure 1:**
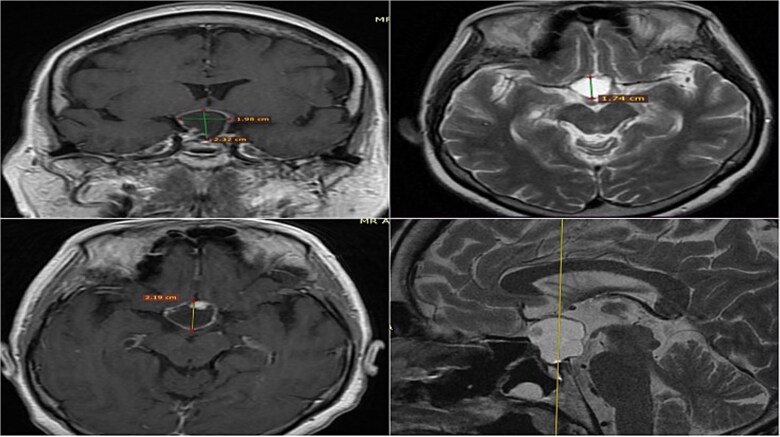
MRI of sellar region tumor in contrasted T2 and T1 sequences in axial, coronal and sagittal sections.

**Figure 2:**
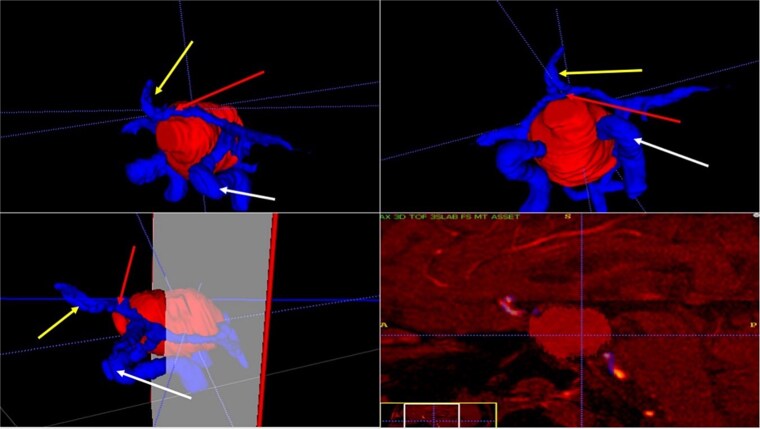
3D reconstruction through manual planimetric segmentation of the tumor and its vascular relationships. White arrow, left internal carotid artery. Yellow arrow, anterior cerebral artery segment A2. Red arrow, anterior communicating artery.

**Figure 3:**
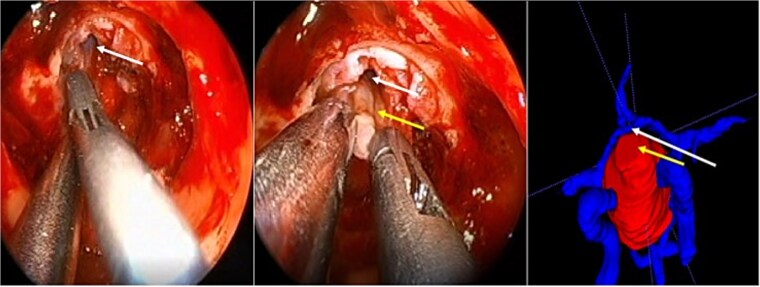
Surgical images. White arrow, anterior communicating artery. Yellow arrow, tumor capsule.

A 71-year-old woman who gradually began with bitemporal hemianopia, progressing to amaurosis of the right eye, adding diabetes insipidus. An MRI study obtained the following images (Fig. 1). 3D reconstruction was performed by manual segmentation to obtain the anatomy of the tumor and its vascular relationships. For this segmentation, it was necessary to splice T2, T1, Contrast, and 3D TOF sequences (Fig. 2). The importance of the reconstruction lies in being able to adequately observe most of the cerebral blood circle and its relationships with the tumor. At the time of carrying out the transnasal resection surgery and keeping in mind the proximity of the anterior communicating artery with the dorso-ventral region of the tumor, the capsule was resected with excessive caution to avoid generating excessive traction and thus avoiding injuries—important vascular (Fig. 3).

### Case 2

**Figure 4:**
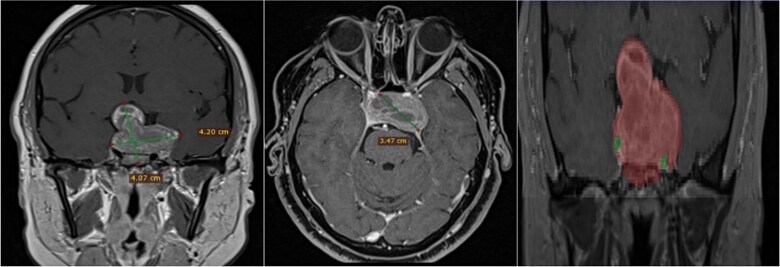
Contrast-enhanced T1 MRI of pituitary marcoadenoma. Multiplanar conventional geometric volumetry.

**Figure 5:**
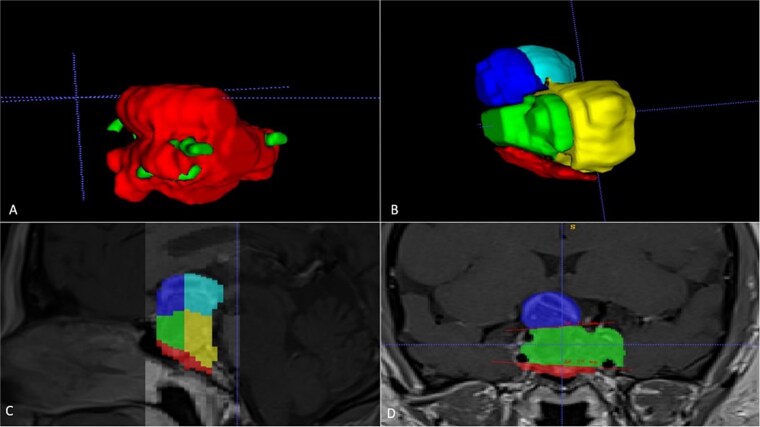
Manual planimetric volumetric segmentation. (A) Calculation of total tumor volume (red) and its relationship with the internal carotid artery (green). (B) Volumetry by zones. (C and D) Areas shown in sagittal and coronal planimetry.

A 42-year-old patient who suddenly presented with headache and visual disturbances. The examination confirmed bitemporal hemianopsia, as well as bilateral papilledema. An MRI study obtained the following sequences: T1, T2, and T1 with contrast (Fig. 4). Manual 3D segmentation was performed to observe the intratumoral components and classify the different anatomical portions of the tumor, as well as its total estimated tumor volume and area (Fig. 5 and Table 2). The importance of reconstruction, in this case, lay in knowing the areas that were possibly not going to be so easily accessible through this endoscopic approach, as well as in calculating the volume of the tumor per segment and the possibility of having residual tumors in less accessible segments.

**Table 2:** Conventional and 3D volumetry, as well as volumetry divided by zones

**Geometric volumetry**	**3D volumetry**	**Zone 1 Red (Intraspenoidal)**	**Zone 2 Green (Intercarotid)**	**Zone 3 Blue stronge (Supracarotid suprasellar)**	**Zone 4 Yellow (Retrocarotid intraselar)**	**Zone 5 Blue clear (Supraselar retrocarotid)**
16 cubic centimeters	21 cubic centimeters	1.7 cubic centimeters	3.7 cubic centimeters	2.3 cubic centimeters	9.7 cubic centimeters	3.6 cubic centimeters

### Case 3

**Figure 6:**
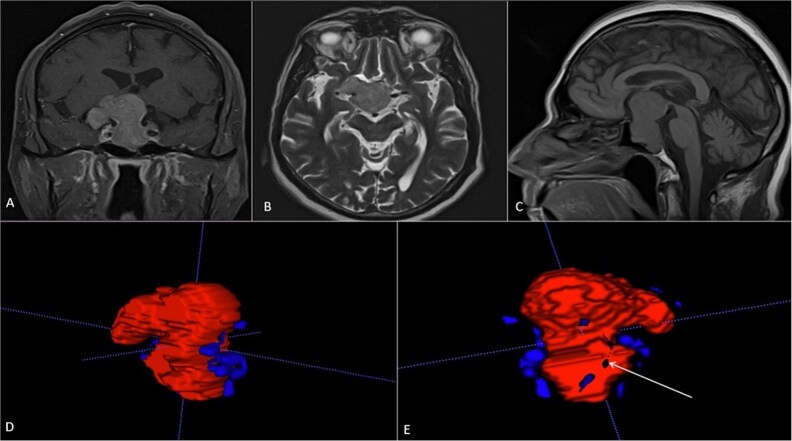
(A, B and C) MRI of pituitary marcoadenoma in coronal, axial and sagittal sections, contrasted T1, T2, and T1 sequences. (D and E) Planimetric segmentation with 3D reconstruction of the basilar and carotid system. White arrow, relationship of the dorsum of the tumor with the basilar artery.

A 68-year-old male patient with an 8-year history of gradual loss of visual acuity. An MRI study was performed with the following sequences: T1, T2, and T1 with contrast. When performing the three-dimensional segmentation, we realized the intimate relationship that the dorsum of the tumor had with the basilar artery; its dolichoectatic course on the dorsal surface of the tumor was what we paid the most attention to and what made us plan a better transnasal approach and avoid the traction of the capsule mainly the dorsal region (Fig. 6).

### Case 4

**Figure 7:**
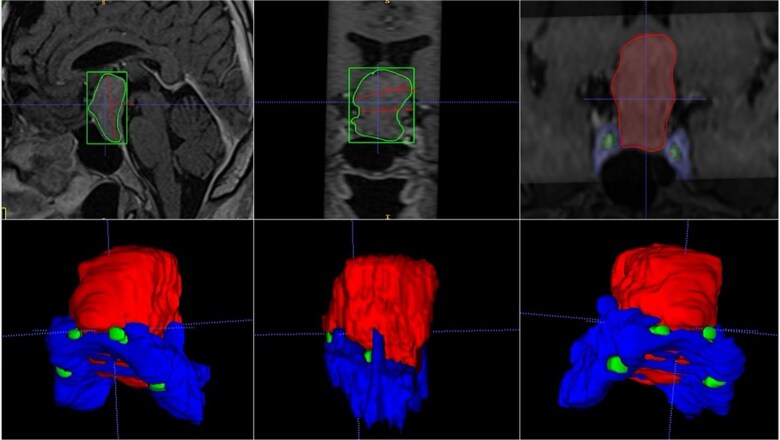
MRI and 3D volumetric segmentation of pituitary macroadenoma. Note the proximity of the internal carotid artery (green) in the most rostral and dorsal region of the tumor (red). Cavernous sinus (blue).

A 42-year-old male patient presented with a history of difficulty seeing in both temporal fields. The examination confirmed said bitemporal hemianopsia. We observed a sellar lesion in the MRI (T1, T2, and T1 sequences with contrast). With the segmentation, we were able to almost reconstruct the cavernous sinus and the internal carotid artery wholly in its intracavernous and clinoid portion and thus take more caution in the most rostral and dorsal region of the tumor since it was the site where both clinoid carotid arteries were closest each other (Fig. 7).

## Discussion

Surgical results in any discipline depend on the skills of the surgeon, his team, and his ability to understand the three-dimensionality of the anatomical region to be operated on [8]. To improve this anatomical understanding, 3D-printed models have been made with the help of segmentation. These models have favored the compression and study of anatomical regions and spaces and have also served as support during the analysis of neurosurgical cases [9, 10]. There are reports where 3D images have led to a successful surgery and better planning, and although the surgeon’s visuospatial orientation requires years of training, 3D volumetry and segmentation have proven to be handy tools that help the development of said visuospatial skills in multiple fields of neurosurgery, such as oncological neurosurgery, vascular neurosurgery, and even functional neurosurgery [11–13]. Without a doubt, neuronavigation has become a valuable tool for the surgeon before and during surgery. However, there are still errors inherent to the system and its operator. Although it has been described that the dual use of neuronavigation and 3D segmentation in pre-surgical planning could reduce these errors [14].

There are automatic segmentations and volumetrics, which have had good performance and reproducibility [15, 16]. However, manual segmentation facilitates anatomical learning during the delineation of normal and pathological structures. Although automatic segmentation tools are helpful because they generally save time, these tools focus only on tumor, vascular, or bone tissue, excluding tissues or areas that, without human intervention, would be more complicated to highlight. With manual segmentation and its respective reconstruction, we can reconstruct all these structures, expanding our 3D vision and anatomical knowledge.

On the other hand, geometric volumetry methods approximately calculate the volume of intracranial lesions using the principal axes of the lesions in planimetric sections; however, these lesions are not spherical but somewhat irregular, so they are measured with a range of essential errors [17–19]. 3D volumetry has even been proven in growth hormone-secreting tumors, where presurgical volumetric measurement, less than two cubic centimeters, presented higher cure rates. Even the more significant the measured tumor volume, the more significant the correlation with higher growth hormone concentrations in the blood [20].

The above is similar to our case number two, where the tumor is quite irregular in conventional ABC/2 volumetry; the real volumetric value of the tumor is underestimated; however, with our manual segmentation, the volumetry can even be measured by zones. In that case, it was important to measure the area where there was a greater possibility of residual tumor and thus be able to correlate it with the possibility of greater rates of total resection.

Although 3D reconstruction images are not new, the imaging studies with which we can make these reconstructions have been improving and moving from low-resolution tomography to magnetic resonance studies in multiple slices and sequences [21]. Although 3D technology has been used for many years in pituitary macro adenomas and sellar tumors, the utilities and applications of this tool have changed over time, from diagnostic methods to surgical events [22, 23]. Therefore, we recommend using these technologies in surgical planning because these could improve surgical care and enhance microsurgical knowledge.

## Conclusion

Imaging studies such as magnetic resonance imaging and tomography, among others, have favored the more detailed study of the anatomical structures in brain tumor pathology. Neuronavigation has become an indispensable tool during surgery and has decreased complications associated with excessive manipulation of brain tumors. However, 3D technology has become a more useful tool in surgical planning because we can visualize the limits of our injury and its relationship with vital structures that are otherwise difficult to imagine when seen in a two-dimensional image. This work exemplifies the usefulness of 3D technology in planning endoscopic endonasal surgeries for the different tumor pathologies of the sellar region. This work demands future studies that compare the use of this technology in the learning and better understanding of microsurgical anatomy and its impact on the skill curve of neurosurgeons in training.
